# COVID-19 health awareness among the United Arab Emirates population

**DOI:** 10.1371/journal.pone.0255408

**Published:** 2021-09-13

**Authors:** Balsam Qubais Saeed, Iffat Elbarazi, Mai Barakat, Ahmed Omer Adrees, Kubais Saeed Fahady

**Affiliations:** 1 College of Medicine, University of Sharjah, Sharjah, United Arab Emirates; 2 Sharjah Institute for Medical Research, University of Sharjah, Sharjah, United Arab Emirates; 3 Institute of Public Health, United Arab Emirates University, Sharjah, United Arab Emirates; 4 Department of biochemistry, Faculty of Veterinary Medicine, Mansoura University, Mansoura Egypt; 5 College of Humanities and Science, Ajman University, Ajman, United Arab Emirates; McGill University, CANADA

## Abstract

In response to the global COVID-19 epidemic, the United Arab Emirates (UAE) government is taking precautionary action to mitigate the spread of the virus. The aim of this study was to evaluate the knowledge and practices toward COVID-19 among the general public in the UAE during the current outbreak. A cross-sectional online survey of 1356 respondents in the UAE was conducted during the epidemic outbreak between 9^th^ to 24^th^ June-2020. The questionnaire consisted of three sections: Socio-demographic, knowledge, practices. Independent-samples t-test, one-way analysis of variance (ANOVA), chi-square and binary logistic regression was used. A p-value of (p < 0.05) was considered statistically significant. The total correct score of knowledge and practice questions was high 85% and 90%, respectively. Male’s sex, other marital status, and illiterate/primary educational levels had a lower level of knowledge and practices than others. Participants aged 18–29 had little higher knowledge than other ages but had a lower level in practices, people who live in Abu Dhabi had better knowledge and practices than other emirates, employed people had a lower level of knowledge but higher in practices. Binary logistic regression analysis presented that females, 18–29 years, and married participants significantly associated with a higher score of knowledge, while female, over 30 years old, the martial status of singles, college-level and higher, unemployed, were significantly associated with high mean practices score. This study provided a full screening of the knowledge and practices among a sample of residents in The UAE toward COVID-19, continuing to implement the health education programs pursued by the UAE is highly important to maintain the appropriate level of awareness among the public.

## 1. Introduction

Coronavirus disease 2019 (COVID-19), is a respiratory tract infection caused by a new strain of coronavirus, which began in Wuhan, China, in December 2019. However, most patients with uncomplicated COVID -19 have mild symptoms such as fever, cough, headache, muscle pain, sore throat, and nasal congestion, some patients develop severe symptoms that need hospital therapy, about 5% of the patients need admission to an intensive care unit [1]. In some cases, Coronavirus 2 (SARS-CoV-2) can lead to acute respiratory distress syndrome and organ failure, such as lung, liver, heart, and kidney [1].

The COVID-19 is rapidly spreading from person to person via respiratory droplets, coughing, or sneezing or contaminated hands of people with the illness. In addition, the disease can spread by touching a surface with large traces of the virus [2]. Many people get COVID-19 if they breathe in droplets from an infected individual with the virus. That is why it is important to stay two meters (6 feet) away from an infected person [2]. Based on the epidemiological reports, the incubation period range of SARS-CoV-2 is 2–14 days, and the virus may be infectious in the patients without symptoms and the infection is more severe in the elderly and people with a chronic health condition [3, 4].

Currently, there is no proven effective treatment against COVID-19, thus, implementing infection control measures and public awareness campaigns to limiting the spread of coronavirus infection is the main way to reduce the prevalence of COVID-19 in the community, especially in middle and low-income countries [5]. In UAE, by11th June of 2020 (when the questionnaire was distributed), there were 41,990 confirmed COVID-19 cases, 26,761 who were fully recovered, 288, deaths while worldwide confirmed cases were 7.41 million and deaths were 418,000 [6]. Currently, managing the crisis depends mainly on people’s adherence to recommended health protection measures. These measures are highly influenced by the knowledge and practices of individuals [5].

Many people are taking preventative measures to protect themselves and their families from COVID-19 all around the world supporting their communities and avoiding the spread of this outbreak. While some people are sharing and apply disappointed information regarding the virus and how to protect against it. Misinformation and an absence of health awareness and information during the COVID-19 outbreak result in individuals not being protected or doing that can harm themselves and others [7].

Public awareness campaigns play an important part in raising the awareness of the public and in drawing their attention to the risks of COVID-19 [8]. The UAE Government handled the situation since the spread of the COVID-19, in March 2020, in a very efficient way and adopted an integrated strategy in maintaining the performance of all sectors in fighting the spread of the disease. Also, it implemented an intensified awareness campaigns on public hygiene and mandatory sterilization supplies in all places to avoid the spread of COVID-19 in public places [3].

With the rapid transmission of the COVID-19 and the lack of consensus on effective medication or cure for the disease, it is mandatory that the public possess the correct knowledge to follow the right practices as a defense line against the disease. Therefore, this study was conducted to evaluate the knowledge and practices toward COVID-19 among the general public in the UAE during the current outbreak of COVID-19. The results of the current study are expected to provide baseline data regarding the level of knowledge of individuals and highlight misperceptions and malpractices related to preventive measures hence better planning for effective awareness campaigns and take the appropriate action from local authorities.

## 2. Material and methods

### Study design and participants

This cross-sectional survey was conducted online and managed by the main author between 9^th^ -June and 24^th^ -June 2020to determine knowledge and practices related to COVID-19 during the current pandemic. An invitation message using WhatsApp with a link to the survey was sent inviting people above the age of 18, from all nationalities, and currently residing in UAE to participate. The professional and personal networks of the researchers were used to reach a high number of participants to complete the survey. Participation in the current study was voluntary and anonymous. Informed consent was sought from all the respondents prior to data collection by sending a standardized general invitation letter with the survey link to accept or decline to participate in the study. The participant who declined consent were not permitted to open the survey and participate in the study, and participants could withdraw from the survey at any time in line with stipulations of the World Medical Association Declaration of Helsinki Ethical principles [9]. The members who clicked on the link were directed to the Google forms and to avoid the missing data, the participants were requested to fill all the questions of the survey or else could not proceed to the next section. The total number of respondents was calculated, and out of 1367 survey received from members, only 1356 participants were included in this study.

### Questionnaire and data collection

A total of 1356 residents from four different emirates in UAE (Abu Dhabi, Dubai, Sharjah, and Ajman) included in this study. The survey was created after a thorough search of the literature, Ministry of Health and Prevention (MOHAP) in the UAE and World Health Organization (WHO) reports [10–12]. The survey was designed in English and Arabic languages in order to encourage the people to participate, and it was reviewed, validated and pilot tested by the research team and 15 individuals using telephone interviews to clarify and correct any question resulting in a few modifications.

The survey questionnaire consisted of an interface page and three main sections with a total number of 37 questions. The interface of the questionnaire included the title, the objective of the study, information on participants’ confidentiality, and instructions to fill in the survey. The three main parts of the questionnaire included: "socio-demographic data", their past COVID-19 testing status and COVID-19 test result. The second part on "knowledge regarding COVID-19" consisted of 17 questions includes: Clinical characteristics of virus (K1-K5), the spread of the virus (K6-K9) prevention (K10-K13) and risk factors (K15-K17). The third part of the questionnaire consisted of 11 questions that investigated "practices of individuals toward COVID-19 outbreak". Each questionnaire took approximately10-15 minutes to answer. The survey is attached as a (S1 Appendix) in this study.

### Data analysis

Data analysis conducted using Statistical Package for Social Software (SPSS) version 22. Scale reliability was performed to ensure data consistency, (Cronbach’s alpha coefficient = 0.729) indicating good consistency. Socio-demographic characteristics frequencies, knowledge, and practices answers along with descriptive statistics were presented in mean ± stander deviation SD, while qualitative data were presented in frequency (number\percent). Participants’ knowledge and practices scores were compared with demographics factories using independent- samples t-test, one-way analysis of variance (ANOVA).

To evaluate the knowledge, respondents were given yes, no, and not sure response options each question, A correct response (yes) of each question assigned 1 score, while assigned 0 scores for incorrect respondent (no and not sure). Total knowledge scores ranged from 0–17. Practice response options of always sometimes and never were assigned 2 scores for always, sometimes 1 score, and never 0 scores. Total practice scores ranged from 0–22.

Pearson’s chi-square as appropriate is used to determine the association, correlation, and homogeneity between variables. Binary logistic regression analyses using backward stepwise method were used to identify factors associated with good, poor knowledge and practices. A p-value of less than 0.05 (< 0.05) was considered statistically significant.

### Ethics approval

The study was approved by the Research Ethics Committee (RIC) at University of Sharjah, UAE. The reference number is REC- 20-05-31-01, as of 14/06/2020.

## 3. Results

### Respondents’ socio-demographic

"Table 1" Presents the socio-demographic data of the participants. Most of participants were females (72%) and (28%) were males. Of the respondents: (47%) were aged between 30–49 years, (39.8%) are aged between (18–29) years, and (13.2%) were between 50-≤65 years. Most of participants are married (59.7%), and more than half of respondents are from Sharjah (57.4%), while other respondents were from Dubai, Ajman, and Abu Dhabi. With regards to their education level, most participants (65%) had a college degree. Moreover, (35.8%) of participants were employed, (40.6%) unemployed, and (23.5%) were students. Almost (19.0%) were Emiratis while, (81.0%) were non-Emiratis. None of the respondents tested positive for COVID-19, 88.1 and 11.9% included those who had tested negatively and those who stated that they did not know the result of the test, respectively.

**Table 1:** Socio-demographic characteristics of participants, UAE (n = 1356).

Demographic factors		Frequency (n)	Percentage (%)	Mean	Std. Deviation
**Gender**	Male	378	27.9	1.7212	0.44856
	Female	978	72.1		
**Age-group (years)**	18–29	540	39.8	1.7338	0.67801
	30–49	637	47		
	50-≤65	179	13.2		
**Marital status**	Single	510	37.6	1.6504	0.52978
	Married	810	59.7		
	Other*	36	2.7		
**Place of residence**	Abu Dhabi	99	7.3	3.2692	0.976
	Ajman	215	15.9		
	Dubai	264	19.5		
	Sharjah	778	57.4		
**Education level**	Illiterate/primary	24	1.8	2.8732	0.62197
	High school/Diploma	287	21.2		
	College level	882	65		
	Postgraduate	163	12		
**Employment status**	Employed	486	35.8	1.8768	0.76087
	Unemployed	551	40.6		
	Student	319	23.5		
**Nationality**	Emirati	258	19	1.8097	0.39266
	Non- Emirati	1098	81		
**COVID-19 test result**	Positive	0	0	2.1195	0.32446
	Negative	1194	88.1		
	I don’t know	162	11.9		

* Other included divorced, and widows.

### Respondents’ knowledge about COVID-19

"Table 2" Presents that the prevalence of good knowledge score of the 17 knowledge questions was (1.61 ± 0.48). The correct answers to the questions were (85%). Most of the participants (90%) answered that COVID-19 caused by a virus, around (89.8%) answered correctly that the incubation period is between 2–14 days. Most participants (95.6%) had knowledge of the main symptoms of COVID-19. About 79.6 and 63.3% of respondence agreed that no vaccine and treatment is available for COVID-19 until now, respectively. About (92.6%) of the participants reported that SARS-CoV-2 spread through contaminated respiratory droplets of infected people, while 82.7% and 85.8% answered that the virus transmitted through the eyes, and via touching contaminated surfaces, respectively. About (67.8%) reported that the person with COVID-19 having no fever can infect other people. Almost (82%) of participants knew that they should wash their hands at least 20 seconds, while around (92.5%) knew that they can use hand sanitizer if soap is not available.

**Table 2:** Descriptive statistics of general knowledge with of participants about COVID-19, UAE (n = 1356).

	Questions		Yes	No	Not sure	Mean	St. Deviation
**K1**	COVD-19 is caused by Virus.	N	**1219**	36	101	1.1755	0.54212
		%	**89.9**	2.7	7.4		
**K2**	Incubation period range of COVID-19 is 2–14 days.	N	**1218**	36	102	1.177	0.54436
		%	**89.8**	2.7	7.5		
**K3**	The main clinical symptoms of COVID-19 are fever, tiredness, dry cough, and breathing difficulty	N	**1296**	24	36	1.0704	0.34407
		%	**95.6**	1.8	2.6		
**K4**	Is there a vaccine of COVID-19?	N	102	**1080**	174	2.0521	0.44795
		%	7.5	**79.6**	12.8		
**K5**	Is there an active treatment for COVID-19?	N	168	**858**	330	2.1167	0.59517
		%	12.4	**63.3**	24.3		
**K6**	Are the COVID-19 spreads via respiratory droplets (from coughing, sneezing) of infected people?	N	**1255**	30	71	1.1262	0.46326
		%	**92.6**	2.2	5.2		
**K7**	Can COVID-19 have transmitted through the eyes, in addition to the nose and mouth?	N	**1122**	109	125	1.2641	0.61486
		%	**82.7**	8	9.2		
**K8**	Can COVID-19 spreads via through touching contaminated surfaces?	N	**1164**	78	114	1.2245	0.58449
		%	**85.8**	5.8	8.4		
**K9**	A person with COVID-19 having no fever cannot infect others.	N	180	**919**	257	2.0565	0.56361
		%	13.3	**67.8**	19		
**K10**	Hand washing should be at least 20 minutes.	N	**1110**	162	84	3.823	0.71335
		%	**81.9**	11.9	6.2		
**K11**	We can use hand sanitizer or disinfectant to clean our hands when water is not available.	N	**1254**	96	6	1.9336	0.26621
		%	**92.5**	7.1	0.4		
**K12**	The minimum distance should you keep it between you and others when go outside is 6 feet (2 meters)?	N	**864**	132	360	1.9292	0.80643
		%	**63.7**	9.7	26.5		
**K13**	To prevent the spread of COVID-19, individuals should avoid going to crowded places if it’s not necessary.	N	**1332**	0	24	1.0352	0.26314
		%	**98.2**	0	1.8		
**K14**	People who have contact with someone infected with the COVID-19 virus should be immediately isolated in a proper place.	N	**1338**	6	12	1.022	0.1979
		%	**98.7**	0.4	0.9		
**K15**	It is not necessary for children and young adults to take measures to prevent the infection by the COVID-19.	N	96	**1206**	54	1.9677	0.3325
		%	7.1	**88.9**	4		
**K16**	The virus may be more dangerous in patients with chronic diseases and elderly.	N	**1344**	0	12	1.0176	0.1869
		%	**99.1**	0	0.9		
**K17**	Smokers are likely to be more vulnerable to COVID-19.	N	**1008**	60	288	1.4666	0.81974
		%	**74.3**	4.4	21.2		
	**Total**		**84.96**			**1.6151**	**0.4874**

The correct responses for each item are bolded.

About (63.7%) successfully identified that the minimum distance that should be maintained between people is 6 feet (2 meters). Nearly all of the respondents (98.2%) agreed that crowded places should be avoided to reduce virus transmission, (98.7%) reported that individuals that contact with person who have COVID-19 positive test should be directly isolated in a suitable place.

Almost (89%) answered necessity for children and young adults take action to prevent their self. Almost all participants (99%) answered that the virus is high risk in patients with chronic illness and elderly, while (74.3%) believed that smokers are likely to be more vulnerable to COVID-19 infection.

### Respondents’ practices toward COVID-19

“Table 3” Presents participants’ practices toward COVID-19. The prevalence of good practice score among people was (1.16±0.47). The table shows that the total correct answer percentage was very high (90.14%). Most of the participants (94.2%) answered that they stay at home and go out only when necessary, almost (96%) started to wash or sanitize their hands regularly, and (96.5%) wear a mask when they go outside home. We found that (94.2%) of respondents keep a 2-meters distance between them and others, while (94.3%) of participants stopped going to crowded places recently. 88.5% participants indicated that they stopped visiting their relatives and friends during the outbreak. The majority of participants (95.6%) stopped hugging and kissing their relatives or friends, while (70.8%) starting to use a credit/debit card for payment. Our finding showed that (88.9%) stopped sharing their food with others, and (94.1%) stopped handshaking in greetings with others. Overall, two-thirds of participants (78.5%) follow regular updates on COVID 19 from various information sources.

**Table 3:** Descriptive statistics of practices of participants towards COVID-19, UAE (n = 1356).

	Questions		Frequency			Mean	St. Deviation
			Always	Sometimes	Never		
**P1**	Do you stay at home and go out only when it is necessary?	N	**1278**	12	66	1.1062	0.43864
		%	**94.2**	0.9	4.9		
**P2**	Have you started to wash or sanitize your hands regularly?	N	**1302**	12	42	1.0708	0.35753
		%	**96**	0.9	3.1		
**P3**	Do you wear a mask when you go outside?	N	**1308**	6	42	1.0664	0.35214
		%	**96.5**	0.4	3.1		
**P4**	Do you keep distance between you and other when you go outside?	N	**1278**	36	42	1.0885	0.37778
		%	**94.2**	2.7	3.1		
**P5**	Did you stop going to crowded places recently?	N	**1285**	42	36	1.0841	0.36083
		%	**94.3**	3.1	2.6		
**P6**	Did you stop visiting your relatives and friends during the outbreak?	N	**1200**	30	126	1.208	0.5923
		%	**88.5**	2.2	9.3		
**P7**	Did you stop kissing your relatives and friends when you meet them?	N	**1296**	12	48	1.0796	0.37974
		%	**95.6**	0.9	3.5		
**P8**	Do you use a credit/debit card or other non-cash modes methods for payment transactions?	N	**960**	180	216	1.4513	0.75275
		%	**70.8**	13.3	15.9		
**P9**	Did you stop sharing your eating utensils and food with others?	N	**1205**	72	79	1.1696	0.5075
		%	**88.9**	5.3	5.8		
**P10**	Did you stop hand shaking with others?	N	**1276**	8	72	1.1121	0.45374
		%	**94.1**	0.6	5.3		
**P11**	Do you follow regular updates on COVID 19?	N	**1065**	123	168	1.3385	0.68706
		%	**78.5**	9.1	12.4		
	**Total**		**90.14**			**1.1613**	**0.4781**

The correct responses for each item are bolded.

### Sources of information on COVID-19

**Fig 1:**
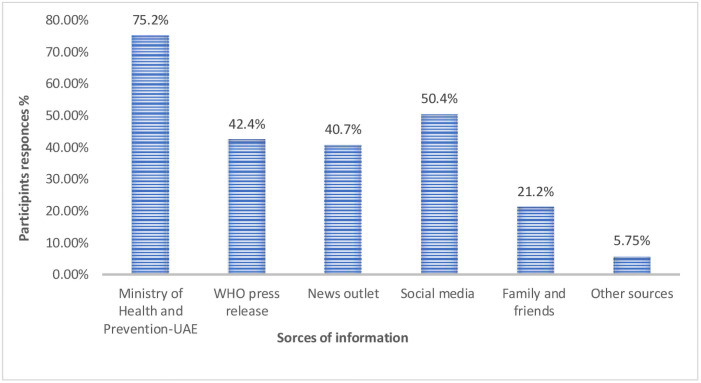
Source of information about COVID-19 among general public in UAE, (n = 1356).

Regarding the sources of information of participants on COVID-19, (75.2%) indicated that their main source of information were official websites and press releases from the Ministry of Health and Prevention (MOHAP) in the UAE. Among them (42.4%) indicated that they were following WHO press releases, while (40.7%) retrieved their information from different news outlet (Newspaper, Television, Radio) and (50.4%) were using social media (Twitter, Facebook, YouTube, WhatsApp, Instagram, and Snapchat). Lastly, 21.2 and 5.75% of participants indicated that their sources of information are mainly from family, friends, and other sources, respectively as displayed in "Fig 1".

### Correlation between socio-demographic characteristics with knowledge (K) and practices (P) score of participants, UAE

"Table 4" Shows that total scores of knowledges (out of 17) significantly differed across gender, marital status, place of residence, education levels and employment status (p <0.05), practices scores (out of 11) also significantly differed across of practice with gender, age-groups, marital status, education levels, and employment status (p <0<0.05) while no significant correlation between place of residence and practices (p = 0.077) was identified.

**Table 4:** Socio-demographic characteristics of participants with knowledge (K) and practices (P) score of COVID-19 by demographic variables of participants, UAE (n = 1356).

**Knowledge**					
**Demographic variables**		**Responses**	**Knowledge score**	**t/f**	**p-value**
		**n (%)**	**(Mean ± SD)**		
**Gender**	**Male**	378(27.9%)	14 ± 2.64	-8.49	0.000
	**Female**	978(72.1%)	**15.09 ± 1.88**		
**Age-group**	**18–29**	540(39.8%)	**14.94±1.44**	5.026	0.007
	**30–49**	637(47%)	14.78± 2.49		
	**50-≤65**	179 (13.2%)	14.35± 2.66		
**Marital status**	**Single**	510(37.6%)	14.65±1.97	36.074	0.000
	**Married**	810(59.7%)	**14.99±2.085**		
	**Others** *	36 (2.7%)	12±4.05		
**Place of residence**	**Abu Dhabi**	99 (7.3%)	**15.33±1.27**	4.966	0.002
	**Ajman**	215(15.9%)	15.10±2.01		
	**Dubai**	264(19.5%)	14.54±2.13		
	**Sharjah**	778(57.4%)	14.71±2.30		
**Education level**	**Illiterate/primary**	24(1.8%)	9.25±2.32	60.928	0.000
	**High school/diploma**	287(21.2%)	14.72±1.83		
	**College level**	882(65%)	14.91±2.073		
	**Postgraduate**	163(12%)	**15.04±2.18**		
**Employment status**	**Employed**	486(35.8%)	14.60±2.30	5.178	0.006
	**Unemployed**	551(40.6%)	**15.01±2.16**		
	**Student**	319(23.5%)	14.67±1.95		
**Practices**					
**Demographic Variable**		**Responses**	**Practice score**	**t/f**	**p-value**
		**n (%)**	**(Mean ± SD)**		
**Gender**	Male	378(27.9%)	19.29±2.58	-7.001	0.000
	Female	978(72.1%)	**20.18±1.87**		
**Age-group**	18–29	540(39.8%)	19.62 ±2.48	12.261	0.000
	30–49	637(47%)	20.05 ±1.92		
	≥ 50	179 (13.2%)	**20.45±1.41**		
**Marital status**	Single	510(37.6%)	19.74 ±2.46	19.422	<0.000
	Married	810(59.7%)	**20.13 ±1.81**		
	Others*	36 (2.7%)	18.1 ±2.59		
**Place of residence**	Abu Dhabi	99 (7.3%)	**20.47±1.88**	2.286	0.077
	Ajman	215(15.9%)	19.87±2.22		
	Dubai	264(19.5%)	19.87±1.79		
	Sharjah	778(57.4%)	19.90± 2.23		
**Education level**	Illiterate/primary	24(1.8%)	14.75±3.26	74.02	0.000
	High school/diploma	287(21.2%)	19.33±2.11		
	College level	882(65%)	**20.29±1.88**		
	Postgraduate	163(12%)	19.80 ±2.00		
**Employment status**	Employed	486(35.8%)	**20.23±1.78**	14.739	0.000
	Unemployed	551(40.6%)	19.98±1.88		
	Student	319(23.5%)	19.41±2.81		

*Other included divorced, and widows.

p<0.05 is significance.

The highest score of knowledge and practices are bolded.

The results score shows that females had a higher knowledge (15.09±1.88) and practice (20.18±1.87) than males, people aged of 18–29 years had a very little higher level of knowledge (14.94±1.44) compared with other age groups, while they had lower level in practice (19.62 ±2.48) than other ages. The knowledge (14.99±2.085) and practices (20.13 ±1.81) of married participants were a little higher than those who identified themselves single, widowed and divorced. Residents of Abu Dhabi showed a better knowledge (15.33±1.27) and practice (20.47±1.88) that other emirates. Participants with lower education showed lower level of knowledge (9.25±2.32) and practice (14.75±3.26) than those with higher education while employed showed lower knowledge (14.60±2.30) than unemployed and students, but they shared higher practices that unemployed and students (20.23±1.78) "Table 4".

### Binary logistic regression associated with the knowledge (K) score (>14.5 and < 14.5) and practices (P) score (>19.5 and < 19.5) as (good and poor) of participants, UAE

"Table 5" presents binary logistic regression analysis on variables significantly correlating with participants knowledge and practice toward COVID-19. The same table presents Odds ratios (ORs) and their 95% confidence intervals (CIs) in a bid to quantify the correlation between factors and the knowledge score (>14 and < 14), and between variables and the practices score (>19 and < 19). The bivariate analysis shows significance when correlated gender, age and marital status and better knowledge. Female gender vs male showed better knowledge (β: 1.146, OR: 3.145, CI:2.235–4.424), age group 18–29 is associated with better knowledge than the age-groups of 30–49 and 50-≤65 respectively (β: -0.917, OR: 0.400, CI: 0.236–0.678), and (β: -1.663, OR:189, CI:0.101–0.355). While being married vs. single participants were significantly associated with the higher mean knowledge (β: 0.559, OR: 1.749, CI:1.017–3.008). Moreover, "Table 5" presents that gender, age, education and employment status also were significantly associated with good practice while marital status was negatively associated with good practice. Female gender vs male (β: 1.619, OR: 5.050, CI: 3.608–7.069), age group of 30–49 and 50-≤65 had higher practice than score than the youngest group (β: 1.134 OR: 3.110, CI:2.019–4.790), (β: 1.981 OR: 7.252, CI: 3.893–13.509) respectively. Marital status of married and others particularly correlated with poor practice score over the single group (β: -1.149 OR:.317, CI:0.185–0.543) and others (β: -2.409 OR:.0.90, CI:0.026–0.309).

**Table 5:** Binary logistic regression analysis on factors significantly associated with mean knowledge (>14 and < 14) and practice (>19 and < 19) as (good and poor), respectively of COVID-19 of the participants, UAE.

**Knowledge (>14 and < 14)**			
**Variable**	**β**	**OR (CI 95%)**	**p-value**
**Gender (female vs. male)**	1.146	3.145 (2.235–4.424)	0.000
**Age (30.49 vs.>18–29)**	-0.917	0.400 (0.236-.678)	0.001
**Age (50 -≤65 vs. 18–29)**	-1.663	0.189 (0.101-.355)	0.000
**Marital status (married vs. single and others)**	0.559	1.749 (1.017–3.008)	0.043
**Practice (>19 and < 19)**			
**Variable**	**β**	**OR (CI 95%)**	**P-value**
**Gender (female vs. male)**	1.619	5.050 (3.608–7.069)	0.000
**Age (30–49 vs. 18–29)**	1.134	3.110 (2.019–4.790)	0.000
**Age (50 -≤65 vs. 18–29)**	1.981	7.252 (3.893–13.509)	0.000
**Marital status (married vs. single)**	-1.149	0.317 (0.185–0.543)	0.000
**Marital status (others vs. single)**	-2.409	0.090 (0.026–0.309)	0.000
**Education level (college level vs. Illiterate/primary)**	2.223	9.231(2.238–38.079)	0.002
**Education level (postgraduate vs. illiterate/primary)**	2.022	7.553(1.885–30.263)	0.004
**Employment (unemployed vs. employed)**	-0.632	0.531(0.364–0.774)	0.001
**Employment (students vs. employed)**	-1.499	0.223(0.132–0.379)	0.000

*Other included divorced, and widows.

p<0.05 is significance.

College-level and postgraduate participants (β: 2.223, OR: 9.231, CI:2.238–38.079) and postgraduate vs illiterate/primary (β: 2.022, OR: 7.553, CI:1.885–30.263), were associated with better practices. Being unemployed or a student showed a poor practice score compare with employed participants (β: -0.632, OR:.531, CI: 0.364–0.774) and (β: -1.499, OR: 0.223, CI:0.132–0.379) respectively.

## 4. Discussion

Our study demonstrated that UAE population and residents have a good to excellent knowledge about COVID-19 causes, risks, precautions, and best practices to avoid infection. It showed that almost 85% of our respondents are knowledgeable and aware of the risk, transmission mode, and hygienic and preventive measures. Being uneducated, unmarried and male were associated with lower knowledge COVID-19 and poorer precautionary measures practices.

To our knowledge, this is the first study to explore COVID-19 knowledge and practices toward the COVID-19 pandemic in the UAE and during the peak of the lockdown. Our study participants were young, non-Emiratis, of female gender with middle to higher education levels which represents the UAE population [13]. This also consistent with similar demographics of participants of surveys collected via online platforms [14]. Similar to our results reported that 86.0% of medical and health sciences students and 80.6% of the university students in UAE were females [15, 16]. This study demonstrated that UAE population and residents have a good to excellent knowledge about COVID-19 and were aware of right practices to avoid the infection and its spread. Our results showed that almost 85% of our respondents were knowledgeable and aware of the risk, transmission mode, and hygienic and preventive measures nearly matching the same percentage by study in China, and another conducted in Jordan among medical students, study among the public in Iraq, and another among the public in Nigeria [4, 11, 17, 18]. A similar trend among respondents in terms of similar gender and education were reported by the study in China [17].

This high and excellent knowledge can be explained by two main reasons: the excellent education programs and campaigns, and action taken by the authorities to contain the COVID-19 spread among the UAE community. Also it can be explained by the media coverage including all media outlets and the impact of the pandemic on social life mandating that people read, listen and get educated about it. However, with all the misconceptions that have been spreading around the virus, our respondents had correct information which indicates that the right message is being spread and it could be again explained by the health authorities ‘actions and education campaigns as well respondents’ education level and access to the right information platforms such as WHO. In contrast to the Jordanian study and Iraq [17, 18], our results showed that the UAE population may depend more on social media, and search engines to get their information about COVID-19, although official websites of health authorities (MOHAP) was the main source of their information on COVID-19 (75.2%) Our results were in the line of other study conducted among students in UAE, as 53.8% and 46.7% were using social media to get the information on COVID-19 [19].

Moreover, this study shows similar findings to that of the Saudi Arabian study [12] in terms of knowledge, practices, and correlated factors such as gender, age, marital status, place of residents, education level, and employment status as being mediators for better knowledge and practices. Comparing the socio-cultural context of both countries, the similar findings and results are very reasonable and are considered normal. Moreover, the efforts of the Ministry of health in both countries were highly intensive in terms of testing and in terms of educating the public and to contain the virus which explains the findings and the similarity in results.

Our study has shown that there was better knowledge and better practices associated with younger ages (below 30), gender (Females), education (higher education), and residing in the capital city of UAE (Abu Dhabi) indicating that more education efforts and more intense health education should be directed toward certain populations like men, lower educated levels and people residing outside AD. The higher significance in terms of the emirate can be explained by the fact that more cases were present in Abu Dhabi lately directing authorities to intensify their education campaigns. Being a female and a mother is expected to show better knowledge and practices in terms of the COVID-19 precautions and preventions, similar to our results, several studies reported that the females had more knowledge and better practices than males [4, 18, 19].

Our results showed, when tested through logistic regression analysis, that, gender, age and marital status were significantly correlated with better knowledge a while being married for married was negatively associated with good practice. Being females, with better education level, and being employed were significantly correlated with higher knowledge and correct practices. Our results were compatible with many studies that showed similar significance in terms of better knowledge among the educated. Better knowledge with higher scores may lead to better practices as shown in this study and as indicated by other studies [10–12]. However, our results did not show any significance when we employed the same factors in our regression. We believe that the significance was not really strong due to the fact again of the intensive education by the authorities and the mandatory hygienic and preventive practices such as washing the hands, using sanitizers, wearing masks, keeping distance, and avoiding touching the face [3]. The findings indicate strongly that awareness campaigns especially social media campaigns and strong health promotions approaches and policies that ensure equity through societies can improve people’s adherence to healthy practices and can induce a positive behavioral change no matter what is their educational background, employment or their social status and gender especially in crisis and in sudden public health events [12, 20, 21]. Health literacy is an important factor in improving people’s adherence to precautions, preventions, and screening. However, with the emerging of this pandemic and during crisis management, improving health literacy will depend mainly on people’s access to knowledge, reading, and the ability to reflect critically on complex health issues [10]. Therefore, the efforts on improving awareness and keep spreading the right message are highly mandatory to ensure healthy practices among the community. The COVID-19 crisis is creating a burden on individuals, communities, and authorities that needs a complex integration of efforts to overcome and to manage this crisis. Studies on COVID-19 have pointed strongly to the mental health impacts and the burden the COVID-19 is creating on public health [22, 23]. In fact, when asked our participants whether they got tested for COVID-19 18.9% stated that they were tested which indicated again the influence of the UAE government efforts of screening [3]. However, what was not surprising that out of the 11.9% that were not positive some declined to declare their results which indicated that there is a new trend of stigma and stereotype that is emerging with the pandemic spread and that needs to be addressed. In fact, studies have shown that COVID-19 is causing anxiety and affecting mental health due to many factors including the COVID-19 positive result stigma [24–26].

## 5. Limitations

This study was done in a very short time and used convenience samples through an online platform that expected that the people with a higher level of education would access and respond to the survey, moreover, it doesn’t give privilege to the uneducated population. Also, the largest percentage of respondents were residents of Sharjah, UAE; thus, the results of this manuscript are not a representation of the total population of the United Arab Emirates. The study dataset was also self-reported, and this practice is subject to courtesy bias or social desirability bias. In addition, knowledge questions are not validated. Another limitation includes the high number of females than males, the possibility of bias being a cross-sectional study, and the possibility of participants looking for answers online.

## 6. Conclusions

This study provided a full screening of the knowledge and practices among a sample of residents in the UAE about COVID-19. Overall, UAE residents showed high levels of knowledge and expected practices, similar to most reports around the world. Obtaining information depends on official news of MOHAP, WHO, and social media. Strategies to keep the community updated and vigilant about precautions should continue and more focus on specific groups like males and people with limited education.

## Supporting information

S1 Appendix Survey study. (PDF)

## Abbreviations

ANOVA: Analysis of variance
COVID-19: Coronavirus disease 2019
K: Knowledge
MOHAP: Ministry of Health and Prevention
P: Practices
SD: Stander deviation
SPSS: Statistical Package for Social Software
UAE: United Arab Emirates
WHO: World Health Organization
