# Correction: COVID-19 health awareness among the United Arab Emirates population

**DOI:** 10.1371/journal.pone.0314212

**Published:** 2024-11-15

**Authors:** Balsam Qubais Saeed, Iffat Elbarazi, Mai Barakat, Ahmed Omer Adrees, Kubais Saeed Fahady

There is an error in the affiliation for author Iffat Elbarazi. The correct affiliation is: Institute of Public Health, United Arab Emirates University, Alain, United Arab Emirates.
