# Supplementary material for: COVID-19 health awareness among the United Arab Emirates population
Source: PLoS One. 2021 Sep 13;16(9):e0255408. doi: 10.1371/journal.pone.0255408 (PMC8437279; doi:10.1371/journal.pone.0255408)
Supplement: S1 Appendix — (PDF) [file pone.0255408.s001.pdf]

UNIVERSITY OF SHARJAH
where civilizations meet

COVID-19 health awareness among the United Arab Emirates Population

You are invited to participate in online survey entitled " COVID-19 health awareness among the United Arab Emirates Population"

The study aims to assess the knowledge and perceptions and health awareness about COVID-19 among the general public in the UAE during the current outbreak.

Your participation in this study is completely voluntary. You may refuse to take part in the research or exit the survey at any time. The researchers will maintain total confidentiality of your responses, and all data will be anonymous without any indication of personal identity. response will help us learn more about the level of health awareness among people in the UAE and assess the positive and negative impacts of this behavior during the COVID-19 pandemic period.

If you have any queries or concern about this research, please contact Dr Balsam Q. Saeed , Assistant Professor in Microbiology , College of Medicine, University of Sharjah, email: bsaeed@sharjah.ac.ae

If you have any ethical issues contact the chair of the Research and Ethics Committee of University of Sharjah, Dr. Suhail Al-Amad at the email: salamad@sharjah.ac.ae

The questionnaire may take 5-10 minutes

Thank you in advance for your participation

ندعوك للمشاركة في استبيان عبر شبكة المعلومات (الانترنت) تحت عنوان " الوعي الصحي حول مرض كوفيد-19 المستجد لدى الافراد في دولة الامارات العربية المتحدة "

إن المشاركة في هذه الدراسة اختيارية بشكل كامل، حيث يمكنك الرفض أو الخروج من الاستبيان في أي وقت. سيحافظ الباحثون على السرية التامة لأجوبتك، وستكون جميع البيانات مجهولة المصدر دون أي إشارة للهوية الشخصية. سوف تساعدنا إجاباتك في معرفة المزيد حول مستوى الوعي الصحي للأفراد في دولة الامارات حول فيروس كوفيد-19 وماهي الاثار الإيجابية والسلبية المترتبة على ذلك خلال الفترة الوبائية.

يمكن ان يستغرق تعبئة الاستبيان 5-10 دقائق.

إذا كان لديك أي استفسار أو ترغب في الحصول على نسخة من نموذج الموافقة، الرجاء التواصل مع:
الدكتورة بلسم قبيس سعيد في قسم العلوم السريرية – كلية الطب – جامعة الشارقة

البريد الالكتروني:

bsaeed@sharjah.ac.ae

الشكر الجزيل لمشاركتكم

Please indicate your willingness to participate in this survey.

- I ACCEPT TO PARTICIPATE IN THIS SURVEY
- I DON'T ACCEPT TO PARTICIPATE IN THIS SURVEY

Please specify

- I took the Coronavirus test
- I didn't take the Coronavirus test

Please specify

Are you COVID-19 positive?

- Yes
- No
- I don't know

Please select the desired language

English

Arabic

Demographic characteristics

1. **What is your gender?**
- Male
- Female

2. **What is your age?**

- 18-29
- 30-49
- 50-≤ 65

3. **What is your marital status?**
- Single
- Married
- Divorced
- Widower

4. **What Emirate do you live in?**
- Abu Dhabi
- Ajman
- Dubai
- Sharjah
- Fujairah
- Ras Al-Khaimah
- Umm Al Quwain

5. **What is your Education level?**
- Illiterate
- Primary
- High school/diploma
- College level
- Postgraduate (Masters/Doctorate)

6. **What is your employment status?**

- Eemployed
- Unemployed
- Student

7. **Nationality**
- Emirati
- Non- Emirati

knowledge about COVID-19

1. **COVD-19 is caused by virus**

- Yes
- No
- Not sure

2. **Incubation period rang of COVID-19 is 2-14 day.**
- Yes
- No
- Not sure

3. **The main clinical symptoms of COVID-19 are fever, tiredness, dry cough, and breathing difficulty.**
- Yes
- No
- Not sure

4. **Is there a vaccine for COVID-19?**
- Yes
- No
- Not sure

5. **Is there an active treatment for COVID-19?**
- Yes
- No
- Not sure

6. **The COVID-19 spreads via respiratory droplets (from coughing, sneezing) of infected people.**
- Yes

- No
- Not sure

7. **Can COVID-19 have transmitted through the eyes, in addition to the nose and mouth?**
- Yes
- No
- Not sure

8. **Can COVID-19 spreads via through touching contaminated surfaces?**
- Yes
- No
- Not sure

9. **A person with COVID-19 having no fever cannot infect others.**
- Yes
- No
- Not sure

10. **Hand washing should be at least 20 minutes.**
- Yes
- No
- Not sure

11. **We can use hand sanitizer or disinfectant to clean our hands when water is not available.**
- Yes
- No
- Not sure

12. **The minimum distance should you keep it between you and others when go outside is 6 feet (2 meters)?**
- Yes
- No
- Not sure

13. **To prevent the spread of COVID-19, individuals should avoid going to crowded places if it's not necessary.**
- Yes
- No
- Not sure

14. People who have contact with someone infected with the COVID-19 virus should be immediately isolated in a proper place.

- Yes
- No
- Not sure

15. It is not necessary for children and young adults to take measures to prevent the infection by the COVID-19.

- Yes
- No
- Not sure

16. The virus may be more dangerous in patients with chronic diseases and elderly.

- Yes
- No
- Not sure

17. Smokers are likely to be more vulnerable to COVID-19.

- Yes
- No
- Not sure

Practices of participants toward COVID-19

1. Do you stay at home and go out only when it is necessary?

- Always
- Sometime
- Never

2. Have you started to wash or sanitize your hands regularly?

- Always
- Sometime
- Never

3. Do you wear a mask when you go outside?

- Always
- Sometime
- Never

4. Do you keep distance between you and other when you go outside?

- Always
- Sometime
- Never

5. Did you stop going to crowded places recently?
- Always
- Sometime
- Never

6. **Did you stop visiting your relatives and friends regularly during the outbreak?**
- Always
- Sometime
- Never

7. Did you stop kissing your relatives and friends when you meet them?
- Always
- Sometime
- Never

8. Do you use a credit/debit card or other non-cash modes methods for payment transactions?
- Always
- Sometime
- Never

9. Did you stop sharing your eating utensils and food with others?
- Always
- Sometime
- Never

10. Did you stop shaking hand shaking?
- Always
- Sometime
- Never

11. Do you follow regular updates on COVID 19?
- Always
- Sometime
- Never

12. Where can you find more information about COVID 19? (You can choose more than answer)
- Ministry of Health and Prevention in the UAE
- World Health Organization press release.
- News outlet (Newspaper, Television, Radio)

- Social media (Twitter, Facebook, YouTube, WhatsApp, Instagram, and Snapchat).
- Family and friends
- Other sources………………….

Thank you for your response
